# Immune Tolerance Induction Using Fetal Directed Placental Injection in Rodent Models: A Murine Model

**DOI:** 10.1371/journal.pone.0123712

**Published:** 2015-04-13

**Authors:** Kei Takahashi, Masayuki Endo, Takekazu Miyoshi, Mitsuhiro Tsuritani, Yukiko Shimazu, Hiroshi Hosoda, Kotaro Saga, Katsuto Tamai, Alan W. Flake, Jun Yoshimatsu, Tadashi Kimura

**Affiliations:** 1 Department of Obstetrics and Gynecology, Osaka University Graduate School of Medicine, Suita, Osaka, Japan; 2 Department of Perinatology and Gynecology, National Cerebral and Cardiovascular Center, Suita, Osaka, Japan; 3 Department of Biochemistry, National Cardiovascular Center Research Institute, Suita, Osaka, Japan; 4 Department of Gene Therapy Science, Osaka University Graduate School of Medicine, Suita, Osaka, Japan; 5 Department of Stem Cell Therapy Science, Osaka University Graduate School of Medicine, Suita, Osaka, Japan; 6 Center for Fetal Diagnosis and Treatment, The Children's Hospital of Philadelphia, Philadelphia, Pennsylvania, United States of America; Medical Faculty, Otto-von-Guericke University Magdeburg, Medical Faculty, GERMANY

## Abstract

**Objectives:**

Induction of the immune response is a major problem in replacement therapies for inherited protein deficiencies. Tolerance created *in utero* can facilitate postnatal treatment. In this study, we aimed to induce immune tolerance towards a foreign protein with early gestational cell transplantation into the chorionic villi under ultrasound guidance in the murine model.

**Methods:**

Pregnant C57BL/6 (B6) mice on day 10 of gestation were anesthetized and imaged by high resolution ultrasound. Murine embryos and their placenta were positioned to get a clear view in B-mode with power mode of the labyrinth, which is the equivalent of chorionic villi in the human. Bone marrow cells (BMCs) from B6-Green Fluorescence Protein (B6GFP) transgenic mice were injected into the fetal side of the placenta which includes the labyrinth with glass microcapillary pipettes. Each fetal mouse received 2 x 10^5^ viable GFP-BMCs. After birth, we evaluated the humoral and cell-mediated immune response against GFP.

**Results:**

Bone marrow transfer into fetal side of placenta efficiently distributed donor cells to the fetal mice. The survival rate of this procedure was 13.5%(5 out of 37). Successful engraftment of the B6-GFP donor skin grafts was observed in all recipient (5 out of 5) mice 6 weeks after birth. Induction of anti-GFP antibodies was completely inhibited. Cytotoxic immune reactivity of thymic cells against cells harboring GFP was suppressed by ELISPOT assay.

**Conclusions:**

In this study, we utilized early gestational placental injection targeting the murine fetus, to transfer donor cells carrying a foreign protein into the fetal circulation. This approach is sufficient to induce both humoral and cell-mediated immune tolerance against the foreign protein.

## Introduction

Induction of immunologic response is a major problem in replacement therapies for inherited disorders such as hemoglobinopathies, immune deficiencies, or certain inborn errors of metabolism. When allogeneic transplantation is performed after birth, intensive immunosuppression and myeloablation is required to avoid rejection or graft versus host disease.

Immune tolerance created by *in utero* exposure to antigen may facilitate postnatal replacement therapies.[1,2] It is well known that under specific circumstances, early gestational exposure to a specific antigen can induce antigen specific tolerance. In humans, the window for tolerance induction is thought to be limited to the first trimester, ending after approximately 14 weeks gestation.[3,4]

Chorionic villus sampling (CVS) is widely utilized for prenatal diagnosis and has been demonstrated to be feasible and safe when performed at 10 to 14 weeks of gestation. Thus, the technique used for CVS is an attractive approach to deliver cells and or foreign antigens to the fetus with appropriate timing to achieve fetal tolerance.

Historically, there have been previous studies utilizing intraplacental bone marrow transplantation in the early gestational mouse model. The classical studies of Fleischman and Mintz [5,6] demonstrated hematopoietic engraftment and chimerism after intraplacental injection of hematopoietic cells, but tolerance was not investigated. However, in those studies, the placenta was blindly injected, and delivery of the cells to the fetal circulation was inconsistent.

In this study, we utilized high-resolution ultrasound guidance in the murine model to inject bone marrow cells expressing a foreign protein (GFP) into the fetal side of the placental circulation, mimicking the CVS procedure. We then analyzed tolerance for the immunogenic GFP protein after birth.

## Methods

### Ethical Statement

All procedures in this study were carried out in strict accordance with the guidelines for animal experimentation from the Animal Research Committee of Osaka University and that of National Cerebral and Cardiovascular Center. The protocol was approved by the Animal Research Committee, Osaka University (Pemit Number: 24-079-018), and National Cerebral and Cardiovascular Center (Permit Number: 13018). All surgery was performed under anesthesia, and all efforts were made to minimize suffering.

### Mouse Recipients and Donors

Accurately time-dated pregnant C57BL/6 mice were used as recipients at embryonic day 10 (E10; 10 days post conception). Donor cells were from C57BL/6TgN(act-EGFP) OsbY01 mice (kindly provided by Dr. Okabe, Osaka University, Genome Information Research Center—referred to as B6GFP in this report) that have been maintained in our breeding colonies. Injected mice were housed in the Laboratory Animal Facility at National Cardiovascular Center Research Institute. The experimental protocols were approved by the Institutional Animal Care and Use Committee at the National Cardiovascular Center Research Institute.

### Preparation of Donor BMCs

Adult GFP^+^ BMCs (B6GFP-BMCs) were isolated from 8 week old B6GFP mice by flushing the tibiae, femurs and iliac bones with Ca/Mg-free phosphate-buffered saline (PBS) using a 26-gauge needle. After filtration through a 40-μm nylon mesh filter, B6GFP-BMCs were centrifuged at 440 x *g* for 5 minutes at 4°C. After the red blood cells were lysed with lysing buffer, the B6GFP-BMCs were counted and suspended in PBS at a density of 4 x 10^7^ cells/ml for injection.

### Intra-Chorionic Villi Injection (ICVI)

**Fig 1:**
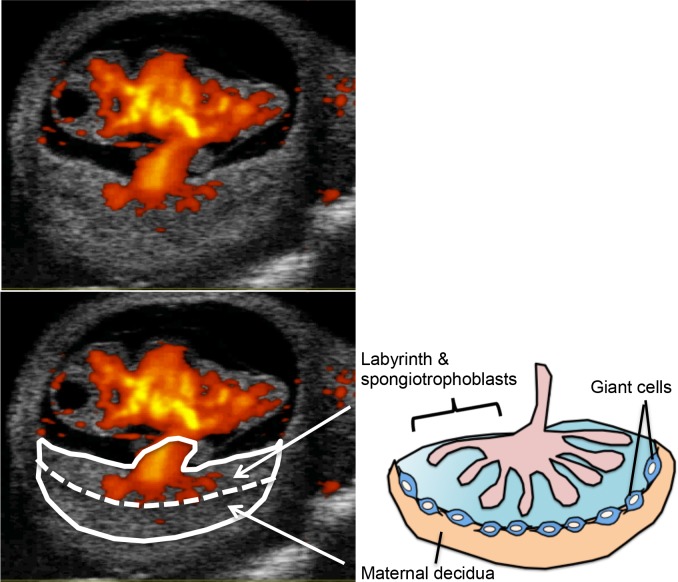
E10 embryo imaged by power Doppler mode. The two layers of the placenta, which consist of the labyrinth and spongiotrophoblasts layer and the maternal decidua. The white dotted line indicates the border between these two layers.

We used an ultrasound-guided injection system (Vevo 2100, VisualSonics, Toronto, Canada) to precisely identify two layers of the murine placenta, which consist of the labyrinth and spongiotrophoblast layer and the maternal decidua (Fig 1). The labyrinth is the area of nutrient and gas exchange between the fetal and maternal circulations. It exists on the fetal side of the placenta and is equivalent to the chorionic villi in the human placenta. Thus, we defined cell transplantation into the labyrinth as intra-chorionic villi injection (ICVI) in this study. Pregnant mice at E10 were anesthetized with isoflorane (3.5% for induction, 2% for maintenance) and placed supine on a platform. A 1-cm ventral midline incision was made through the skin, abdominal wall, and peritoneum. One horn of the uterus was exteriorized and covered in pre-warmed sterile ultrasound gel (Aquasonic, Parker Laboratories, Fairfield, NJ). The fetal mice were scanned using an 80 MHz probe. Pulled and beveled glass microcapillary pipettes (diameter, 75μm) were loaded with B6GFP bone marrow cells at the beginning of each procedure. The fetal mice and placenta were positioned to get clear views of the fetal side of placenta in B-mode with power mode (Fig 1). Under two-dimensional visualization, the micropipette tip was physically inserted into the labyrinth on the fetal side of the placenta and 2 x 10^5^ viable B6GFP-BMCs in a volume of 5μl was injected into each fetus. A new fetus was positioned and the procedure was repeated. After injections, the fetal mice were placed back into the abdominal cavity and the abdominal incision of the dam was closed with 3–0 vicryl continuous suture. The overall time of the procedure was limited to 30 min, from incision until closure.

### Fluorescence Stereoscopic Microscopy

**Fig 2:**
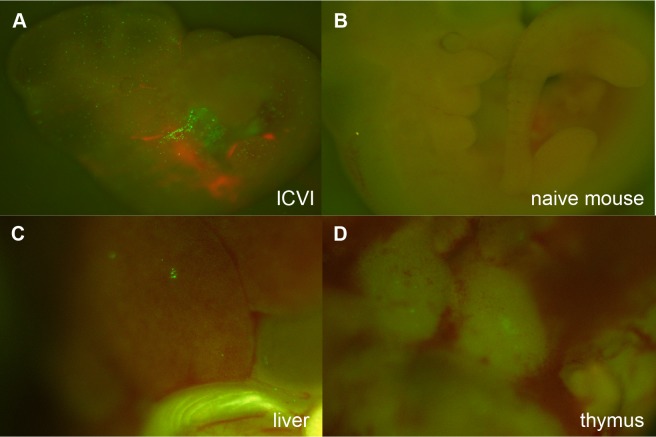
Successful delivery of GFP-BMCs to mouse embryos by ICVI at E10. A: GFP positive cells were detected in the fetal body and heart at 30 minutes after injection (x10). B: Naïve fetus at E10 (x10). C-D: E17 mouse liver and thymus at 1 week after ICVI (x40).

The whole embryo and dissected organs were analyzed under a fluorescence stereoscopic microscope (Leica Microsystems AG, Wetzlar, Germany) and GFP filters to detect migrated GFP positive cells, 30 minutes, 1 week (Fig 2), and 7–9 weeks after ICVI.

### Flow cytometry

Chimerism of bone marrow cells and blood of 6–8 weeks old injected mice was analyzed with a flow cytometer (FACS CantoⅡ, BD, NJ, USA) by detecting GFP+ cells.

### Skin Graft

Full thickness tail skin from B6GFP mice isolated by excision under anesthesia was cut into 10 x 10-mm squares. The cut tail skin was engrafted onto the back of recipient mice at 6 weeks after birth just above the muscular fascia and secured with a bandage for 7 days. Transplanted GFP-skin graft integrity was evaluated under UV light at 7 weeks after skin grafting.

### Measurement of Anti GFP Antibody Generation

At 4 weeks after GFP skin grafting, a blood sample was collected from each animal by tail snip and stored at -20°C. Recombinant GFP was diluted to a working concentration of 0.2 μg/ml in PBS, and 96-well microtiter plates were coated with 100 μl of the diluted GFP solution per well and incubated at 4°C overnight. The plates were rinsed twice with washing buffer (0.05% Tween 20 in PBS), and 200 μl of blocking buffer (1% BSA in PBS) was then added to each well, and the plates were incubated at room temperature for 2 hours. The whole blood harvested from the mice grafted with B6GFP skin was diluted 1:200 in PBS, and 100 μl per well was added for a 1 hour incubation at room temperature. After rinsing with washing buffer, 100 μl of secondary antibody (anti-mouse IgG, horseradish peroxidase, diluted 1:2500 in PBST) was added to each well for 1 hour. The microplates were washed and the levels of antibody generated against GFP were measured with a microplate reader (Berthold Japan K.K., Tokyo, Japan). The methods of skin grafting and measurement of anti-GFP antibody generation were described in detail previously by Chino et al. [7].

### Cytotoxic T-Cell (CTL) Assay against GFP

CTL activity was evaluated by enzyme-linked immunospot assay (ELISPOT). ICVI mice and naïve mice were immunized by intraperitoneal injection with 100 μL of B6GFP-splenocytes as immunogen at both 10 and 11 weeks after birth. Two weeks after the last immunization, mice were anesthetized and splenocytes harvested from isolated spleens by passage through a sterile strainer. The splenocytes were then sedimented by centrifugation at 440 x *g* for 10 minutes and red blood cells were depleted using ACK buffer for 10 minutes. The B6GFP- BMCs were harvested, sedimented by centrifugation at 440 x *g* for 5 minutes at room temperature, resuspended in RPMI 1640, and incubated with Mitomycin C for 20 minutes. Finally, isolated splenocytes (5 x 106/ml) were co-cultured with B6GFP-BMCs (5 x 105/ml) in 58-cm2 BD Falcon dishes with rhIL-2 at 37°C. After sensitization by the B6GFP- BMCs (the stimulator) in vitro for 4 days, the effector splenocytes and B6GFP-BMCs were seeded onto 96-well tissue culture plates (Millipore Ireland B.V.,Germany) coated with anti-IFN-γ specific primary antibodies, at 37°C for 48 hours. The secreted IFN-γ was detected as spots by staining with phosphatase-labeled secondary antibodies and BCIP/NBT solution. The number of spots in each well were counted.

### Statistical analysis

Data are graphically represented as the mean of the respective group plus or minus SEM. Statistical comparisons between groups were performed using a Kruskal-Wallis test and either a Student-t test or a Mann-Whitney U test. Calculation of P value was performed using Microsoft Excel software (Redmond. WA).

## Results

### Ultrasonographic view of the placenta

Pregnant mice were imaged by high resolution ultrasound at E10. When the probe was placed on the surface of the uterus, the fetus, placenta, and umbilical cord could be readily seen. Fetal mice at E10 correspond to Carnegie stage 10, as documented by closure of the posterior neuropore. The umbilical cord was seen inserting into the middle of each placenta. With power Doppler mode, the two layers of the placenta were clearly distinguished; one included the labyrinth and spongiotrophoblast, and the other was the maternal decidua layer. The labyrinth corresponded to the chorionic villi and we could detect fetal blood flow to the umbilical cord with power Doppler mode (Fig 1). The labyrinth was the target site for intra-chorionic villi injection (ICVI). During successful ICVI, the injectate could be observed entering into the fetal circulation through the umbilical cord.

### Survival rate after ICVI

A total of 37 fetal mice from 4 pregnant dams were injected with B6GFP-BMCs on E 10. Survival rate assessed at birth of this procedure was 13.5% (5 out of 37).

### ICVI distributes donor cells to the fetus

To assess the accessibility of the fetal circulation by ICVI at E10, we analyzed the B6GFP cell injected fetal mice under fluorescence stereomicroscopy. Within 30 minutes after successful ICVI, distribution of the injected BMCs to the embryonic yolk sac and fetal circulation was confirmed by observing GFP+ cells on the surface of the yolk sac and in the fetal heart (Fig 2A). At E17, 7 days after ICVI, there were fewer cells detectable, however, GFP^+^ cells could still be detected in the skin, liver, spleen and thymus (Fig 2C and 2D).

We also analyzed recipient mice at 6–8 weeks of age. However, GFP^+^ cells could not detected in the skin, liver, spleen and thymus by fluorescence stereomicroscopy, or in the bone marrow and blood by flow cytometry. (data not shown)

### Humoral and Cellular Immune Tolerance Induction against GFP by ICVI

**Fig 3:**
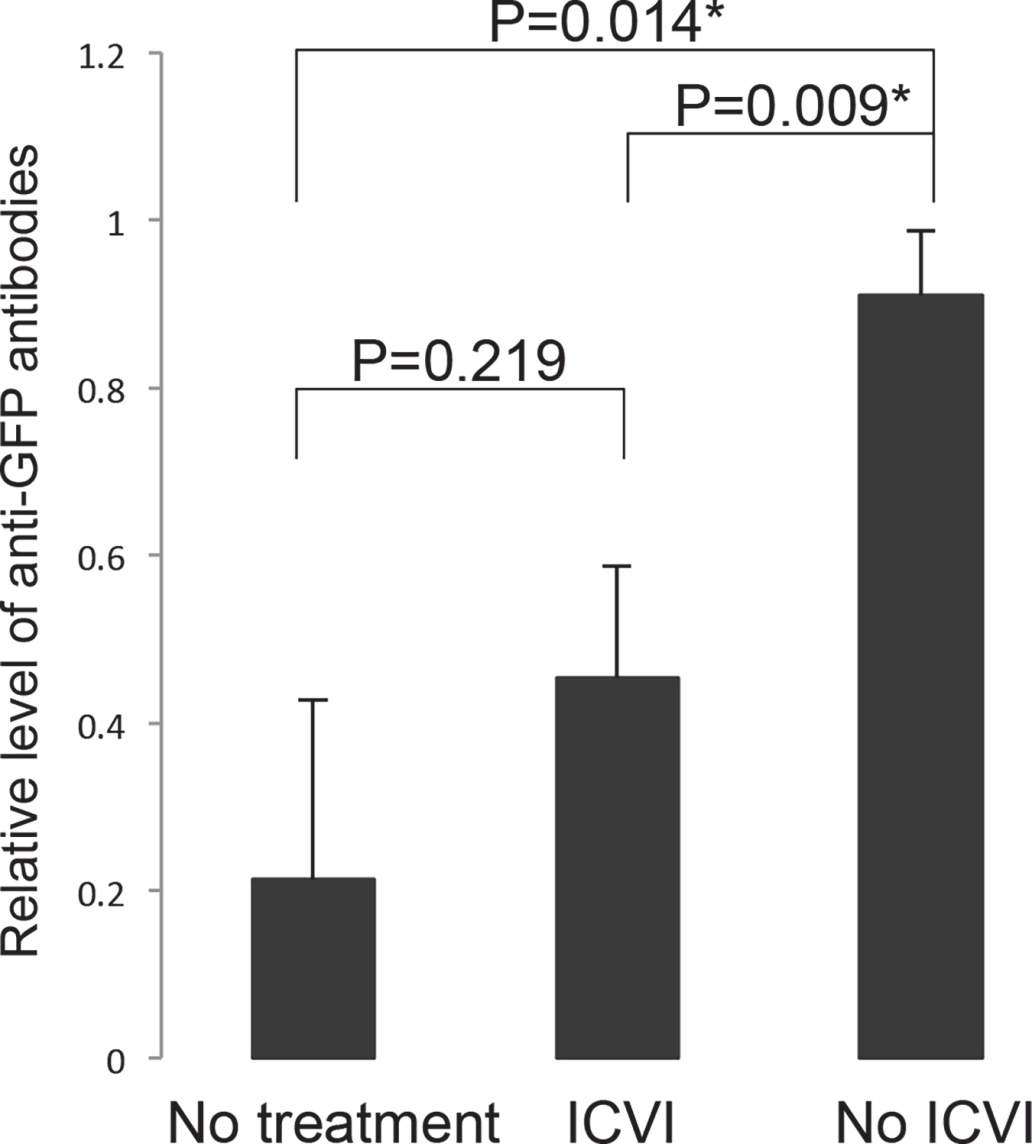
Generation of antibodies against GFP after GFP-skin grafting. Antibodies against GFP were measured using ELISA 4 weeks after skin grafting. Non ICVI control mice (N = 5) produced significant amounts of antibodies, whereas ICVI mice (N = 5) did not. The no treatment group consisted of naïve mice that did not undergo ICVI or skin grafting as a negative control (N = 4). *Statistical significance was assessed if P<0.0167 with Bonferroni correction. P = 0.008 from Kruscal-Wallis test indicating a global difference.

**Fig 4:**
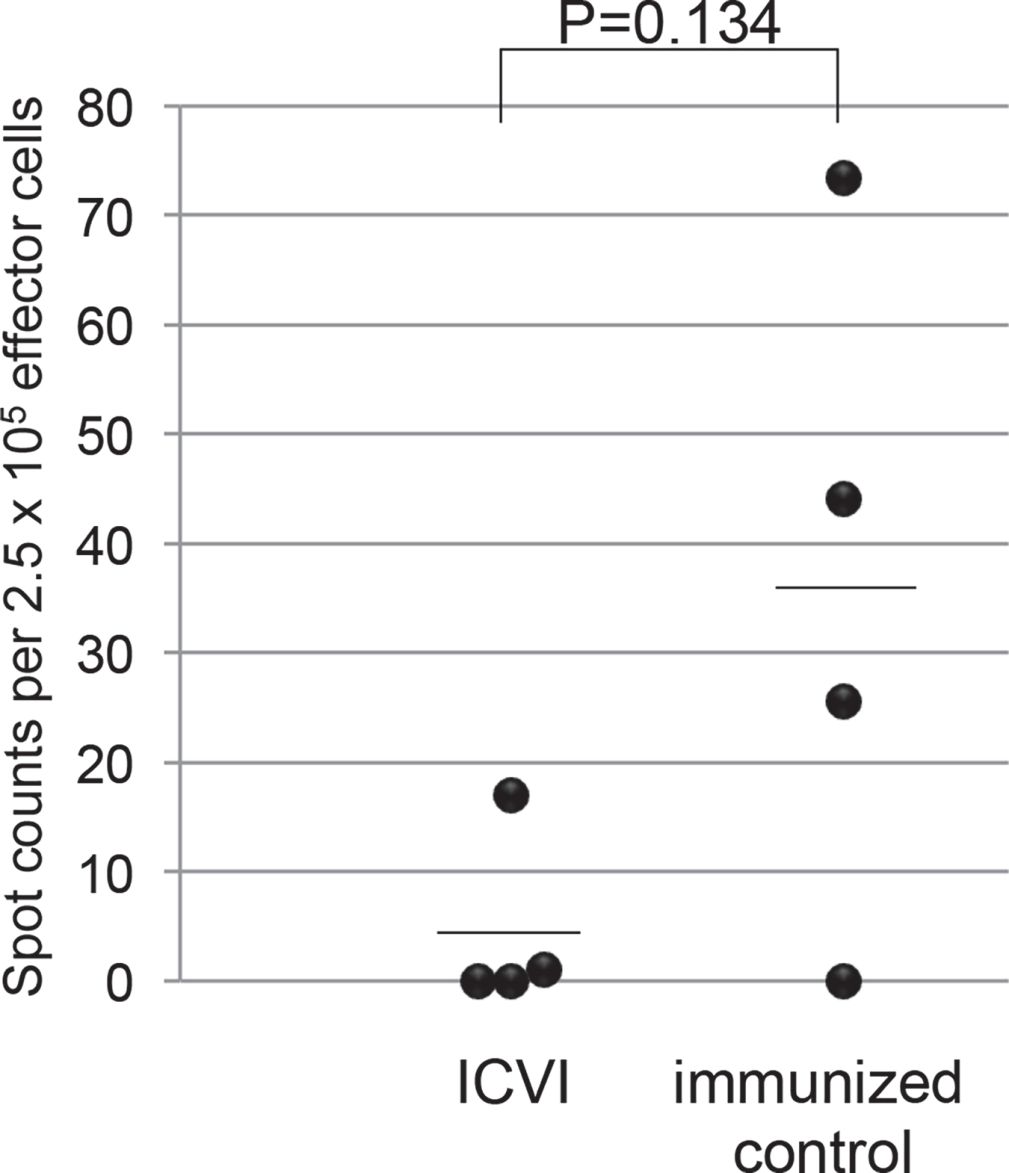
Cytotoxic T cell assay against GFP in ICVI mice (N = 4) and GFP-immunized mice (N = 4). CTL reactivity against GFP-BMCs were measured using ELISPOT which Indicated spot counts per 2.5 x 10^5^ effector cells. ICVI mice had a tendency to generate less response than immunized controls (P = 0.13).

Previous studies have indicated that GFP protein is an immunogenic antigen that evokes both cellular and humoral immune response in mice. We evaluated whether ICVI of congenic B6GFP-BMCs can induce immune tolerance against GFP. First, ICVI mice that received 2 x 10^5^ congenic B6GFP-BMCs were transplanted with the skin from a congenic B6GFP-transgenic mouse. Engrafted GFP-skin persisted at 7 weeks after grafting in all the mice undergoing ICVI (5 out of 5, data was not shown) whereas, all skin grafts were rejected in naïve mice within 8 weeks (N = 8). Induction of anti-GFP antibodies, which was observed in all naive mice receiving GFP skin grafts, was completely inhibited in the mice undergoing ICVI (Fig 3, P = 0.009). Furthermore, cytotoxic immune reactivity of host T-cells against cells expressing GFP was reduced in mice undergoing ICVI as assessed by the ELISPOT assay (Fig 4, P = 0.13). These data suggested that ICVI with congenic B6GFP-BMCs is sufficient to induce both humoral and cellular immune tolerance against GFP.

## Discussion

In this study, foreign protein-expressing bone marrow cells were transplanted into fetal mice by intra-placental injection at a very early gestational stage using an ultrasound-guided injection system. This mimics the clinical technique of CVS which is the earliest fetal intervention in humans. The high-resolution ultrasound system allowed the fetal side of the placenta, specifically the labyrinth, to be clearly distinguished from the maternal decidua. This allowed accurate injection of BMCs which subsequently migrated into and engrafted the fetus. The engrafted GFP^+^BMCs were able to induce immune tolerance against GFP. Thus, we demonstrated that antigen exposure using the ICVI technique is sufficient to induce antigen-specific immune tolerance in utero.

It is well known that exposure to foreign antigen during the preimmune stage of fetal development can establish antigen-specific immune tolerance. There have been several modes of antigen exposure utilized for delivering donor cells or genes during early gestation in animal models. These include intravascular[7,8], intracardiac[9], intraperitoneal[10,11], intraplacental[5,6], and intraamniotic injection.[12] Because of technical limitations, most of the studies were performed at later gestational stages than our study. However, the human fetal immune system begins to develop from the late 1^st^ trimester and is completely established during fetal life. Thus, to induce immune tolerance in humans, it may be advantage to deliver the foreign antigen during the 1^st^ trimester. The only fetal intervention that is clinically applied during the first trimester, is chorionic villous sampling, referred as CVS.

Fleischman et al. used the intraplacental route for injection of cells on day 11 of gestation in the rodent model in 1979.[5] They demonstrated that efficient wild type donor cell chimerism was achieved by intra-placental injection in homozygous anemic mice. However, they inserted micropipettes through the uterine wall into each placenta directly under direct vision, and therefore could not distinguish between the fetal or maternal side of the placenta. Thus, injected cells could enter either the maternal or fetal circulations. The advent of high resolution ultrasound makes it possible to directly image the two sides of the circulation in the murine placenta allowing us in this study to precisely transplant donor cells into the fetal side of the circulation. While the technique is similar to CVS, cells are injected rather than withdrawn potentially converting the technique from a diagnostic endeavor to a therapeutic one.

This implications of this study are limited by the differences in the structure of the placenta and the development of immune system between the mouse and human. In mice, the placenta consists of 3 layers, which are the fetal labyrinth, the spongiotrophoblast layer, and the maternal decidua. The former two layers belong to the fetal side of placenta. In the human, the fetal blood vessels are covered by a layer of cytotrophoblast cells and an adjacent layer of multinucleated syncytiotrophoblast. These structures, called placental villi, are bathed in maternal blood.[13,14] Because of this structural difference, it is unknown whether cells or other antigens transplanted into the human placenta will migrate into the fetal circulation as efficiently as observed in this murine study.

Development of the immune system also differs between humans and mice. Some form of immune tolerance can be induced with exposure to foreign antigen as late as 1 week after birth in mice.[15–17] However, in the human both T cells and B cells begin to develop from 10 weeks gestation with emergence of mature T-cells from the fetal thymus after 13–14 weeks gestation.[3,4] Thus, it may be necessary to utilize ICVI during the 1^st^ trimester, as proven feasible by CVS, to enhance the probability of induction of immune tolerance.

Low fetal survival rate after ICVI was also of concern in our study. Each fetal mouse received 2 x 10^5^ viable B6GFP-BMCs with a volume of 5μl, and the survival rate of this procedure was 13.5%(5 out of 37). In some fetal mice, bleeding was observed by ultrasound during the injection. This is probably related to the volume of injectate and we anticipate improvements in survival with further application of this technique.

In conclusion, we utilized a technique similar to CVS to transfer donor cells carrying a foreign protein into the fetal side of the placenta in the murine model. This approach proved sufficient to induce both humoral and cell-mediated immune tolerance against the foreign protein.

## References

[1] Mattar CN, Biswas A, Choolani M, Chan JK. The case for intrauterine stem cell transplantation. Best Practice & Research Clinical Obstetrics and Gynaecology. 2012 10;26(5):683–95. 22809469 10.1016/j.bpobgyn.2012.06.005

[2] Nijagal A, Flake AW, MacKenzie TC. In utero hematopoietic cell transplantation for the treatment of congenital anomalies. Clin Perinatol. 2012 6;39(2):301–10. 10.1016/j.clp.2012.04.004 22682381

[3] Whitelaw A, Parkin J. Development of immunity. Br Med Bull. Oxford University Press; 1988 1 1;44(4):1037–51. 3076825 10.1093/oxfordjournals.bmb.a072288

[4] Palmer AC. Nutritionally mediated programming of the developing immune system. Adv Nutr. American Society for Nutrition; 2011 9;2(5):377–95. 10.3945/an.111.000570 22332080 PMC3183589

[5] Fleischman RA, Mintz B. Prevention of genetic anemias in mice by microinjection of normal hematopoietic stem cells into the fetal placenta. PNAS. National Acad Sciences; 1979 11 1;76(11):5736–40. 42904 10.1073/pnas.76.11.5736 PMC411725

[6] Aboussaouira T, Gerard A, Gerard H. Effect of in utero infusion route on lymphocyte distribution in fetal rat tissues. Fetal Diagn Ther. 1998 7;13(4):216–22. 9784641 10.1159/000020841

[7] Chino T, Tamai K, Yamazaki T, Otsuru S, Kikuchi Y, Nimura K, et al Bone marrow cell transfer into fetal circulation can ameliorate genetic skin diseases by providing fibroblasts to the skin and inducing immune tolerance. Am J Pathol. 2008 9;173(3):803–14. 10.2353/ajpath.2008.070977 18688022 PMC2527073

[8] Peranteau WH, Endo M, Adibe OO, Merchant A, Zoltick PW, Flake AW. CD26 inhibition enhances allogeneic donor-cell homing and engraftment after in utero hematopoietic-cell transplantation. Blood. 2006 12 15;108(13):4268–74. 16954501 10.1182/blood-2006-04-018986 PMC1895454

[9] Christensen G, Minamisawa S, Gruber PJ, Wang Y. High-efficiency, long-term cardiac expression of foreign genes in living mouse embryos and neonates. Circulation. 2000. 10.1161/01.cir.101.2.178 10637206

[10] Shaaban AF, Kim HB, Gaur L, Liechty KW, Flake AW. Prenatal transplantation of cytokine-stimulated marrow improves early chimerism in a resistant strain combination but results in poor long-term engraftment. Exp Hematol. 2006 9;34(9):1278–87. 16939821 10.1016/j.exphem.2006.05.007 PMC3096442

[11] Vanleene M, Saldanha Z, Cloyd KL, Jell G, Bou-Gharios G, Bassett JHD, et al Transplantation of human fetal blood stem cells in the osteogenesis imperfecta mouse leads to improvement in multiscale tissue properties. Blood. 2011 1 20;117(3):1053–60. 10.1182/blood-2010-05-287565 21088133

[12] Endoh M, Koibuchi N, Sato M, Morishita R, Kanzaki T, Murata Y, et al Fetal gene transfer by intrauterine injection with microbubble-enhanced ultrasound. Mol Ther. 2002 5;5(5 Pt 1):501–8. 11991740 10.1006/mthe.2002.0577

[13] Rossant J, Cross JC. Placental development: lessons from mouse mutants. Nature Reviews Genetics. 2001;2(7):538–48. 11433360 10.1038/35080570

[14] Watson ED, Cross JC. Development of structures and transport functions in the mouse placenta. Physiology (Bethesda). 2005 6;20(3):180–93. 15888575 10.1152/physiol.00001.2005

[15] Billingham RE, Brent L, Medawar PB. Actively acquired tolerance of foreign cells. Nature; 1953. 10.1038/172603a0 13099277

[16] Soper BW, Lessard MD, Jude CD, Schuldt AJT, Bunte RM, Barker JE. Successful Allogeneic Neonatal Bone Marrow Transplantation Devoid of Myeloablation Requires Costimulatory Blockade. J Immunol. American Association of Immunologists; 2003 9 15;171(6):3270–7. 12960357 10.4049/jimmunol.171.6.3270

[17] Bruscia EM, Ziegler EC, Price JE, Weiner S, Egan ME, Krause DS. Engraftment of Donor-Derived Epithelial Cells in Multiple Organs Following Bone Marrow Transplantation into Newborn Mice. Stem Cells. 2006 10;24(10):2299–308. 16794262 10.1634/stemcells.2006-0166

